# Serum long non‐coding RNA LINC00887 as a potential biomarker for diagnosis of renal cell carcinoma

**DOI:** 10.1002/2211-5463.12930

**Published:** 2020-07-27

**Authors:** Junjie Xie, Yu Zhong, Rong Chen, Gang Li, Yongwen Luo, Jintao Yang, Zhongwei Sun, Yanzhong Liu, Peipei Liu, Na Wang, Jiaqi An, Chao Li, Yang Song

**Affiliations:** ^1^ Department of Urology Eighth Medical Center, Chinese PLA General Hospital Beijing China; ^2^ Department of Urology 958th Hospital of PLA Southwest Hospital Army Medical University Chongqing China; ^3^ Center of Clinical Laboratory First Medical Center, Chinese PLA General Hospital Beijing China; ^4^ Department of Urology Zhongnan Hospital of Wuhan University Wuhan China; ^5^ Department of Urology 967th Hospital of PLA Dalian China; ^6^ Outpatient Comprehensive Treatment Area First Medical Center, Chinese PLA General Hospital Beijing China; ^7^ Shandong First Medical University Shandong China

**Keywords:** biomarker, cell proliferation, LINC00887, renal cell carcinoma

## Abstract

The identification of non‐invasive biomarkers for the detection of renal cell carcinoma (RCC) in early‐stage patients may help improve disease outcome. Certain long non‐coding RNAs (lncRNAs) have been reported to be possible biomarkers for the diagnosis and prognosis of cancer. Here, we examined the suitability of the lncRNA LINC00887 as a potential biomarker for RCC because its expression has been shown to be elevated in RCC tissue versus normal tissue in the Gene Expression Profiling Interactive Analysis (GEPIA) database. We found that LINC00887 expression is significantly increased in early‐stage RCC tissues and the serum of early‐stage RCC patients compared to matched normal tissues and the serum of healthy subjects, respectively. We also demonstrated that elevated serum LINC00887 is generated from the tumor tissues of RCC patients. Moreover, a receiver operating characteristic (ROC) curve was generated to analyze the diagnostic value of serum LINC00887. The area under the ROC cure differentiating early‐stage RCC patients from healthy subjects was 0.8001, with a sensitivity of 71.05% and a specificity of 89.87%. Furthermore, we found that LINC00887 promotes RCC cell proliferation *in vitro*. Taken together, our findings suggest that a serum LINC00887 signature is associated with RCC cell proliferation and may be a potential biomarker for the detection of early‐stage RCC.

## Graphical abstract

LINC00887, a long non‐coding RNA, is overexpressed in renal cell carcinoma (RCC) tissue and the sera of RCC patients. High levels of LINC00887 in sera are associated with early‐stage RCC and poor survival of RCC patients, and it also promotes RCC cell proliferation.

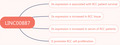

**Abbreviations**
GEPIA: Gene Expression Profiling Interactive Analysis
lncRNA: long non‐coding RNA
qRT‐PCR: quantitative reverse transcriptase‐PCR
RCC: renal cell carcinoma
ROC: receiver operating characteristic

According to new cases and deaths for 36 cancers and all cancers combined in 2018, the incidence of kidney cancer ranks 16th, including 403 262 new cases and 175 098 deaths during the year [1]. It has estimated that 73 750 new cases of cancer of the kidney and renal pelvis and 14 830 cancer‐related deaths will occur in the USA in 2020 [2]. Renal cell carcinoma (RCC) is the most common type of kidney cancer, for which metastasis is the leading cause of kidney cancer‐related deaths. The 5‐year survival rate of patients with metastatic RCC is < 10% [3] However, most patients have developed an advanced‐stage RCC when they are first diagnosed in hospital [4]. Tumor screening using non‐invasive biomarkers may idenitfy patients with early‐stage RCC and therefore improve their outcome.

Long non‐coding RNAs (lncRNAs) are a class of single RNAs of more than 200 nucleotides in length and they do not encode protein [5]. In recent years, lncRNAs have been shown to play important roles in the development of almost all types of cancer [6], including RCC [7]. lncRNA expression presents an organ‐specific characteristic (lncBook; https://bigd.big.ac.cn/lncbook/index), suggesting that lncRNA expression signature may serve as a specific biomarker for the diagnosis or/and prognosis of a disease. Some lncRNAs have been considered to be novel therapeutic targets for cancer [8]. However, a large number of lncRNAs have still not been identified, and their roles and mechanisms in RCC development remain unclear. After analyses of the Gene Expression Profiling Interactive Analysis (GEPIA) data, we focused on an lncRNA, LINC00887, for which the expression is markedly increased in RCC tissue versus matched normal tissue (http://gepia.cancer‐pku.cn/detail.php). Yu *et al*. [9] first reported that LINC00887 expression was increased in RCC tissue versus normal tissue using an lncRNA microarray. However, its exact expression and potential role in RCC remain largely unknown.

In the present study, we found that LINC00887 expression was increased in RCC tissues compared to matched normal tissues, and also increased in the serum of RCC patients versus that of healthy subjects; Receiver operating characteristic (ROC) curve analysis revealed that LINC00887 expression signature in serum had an AUC of 0.8001 with respect to differentiating T1/T2‐stage RCC patients from healthy subjects, and an AUC of 0.8308 with respect to differentiating non‐metastatic RCC patients from healthy subjects; LINC00887 promoted RCC cell proliferation *in vitro*. Our findings indicate that LINC00887 may be a potential biomarker and a therapeutic target for RCC.

## Materials and methods

### Patients and samples

In total, 114 patients with RCC and 79 healthy subjects were enrolled in the present study. In addition, 79 pairs of RCC and normal tissues were collected from the patients (Table 1). The tissues were cut into ~0.5 × 0.5 cm^2^ and immediately stored in nitrogen liquid. Tissue with a distant of more than 3 cm from the tumor was considered normal. All tissues were collected within 10 min after operation and stored in nitrogen liquid. In total, 228 serum samples were collected from the patients and healthy subjects, among which 35 serum samples were obtained from 35 patients who had undergone surgery and went to hospital for review 3 months later. The collection of serum samples was followed by two steps to prevent circulating cell contamination: (a) centrifugation of whole blood at 1000 ***g*** at room temperature; (b) transfer of serum to a fresh tube and centrifugation at 12 000 ***g*** at room temperature. Seventy‐nine serum samples serving as normal controls were collected from the healthy subjects who had taken a physical exam and were confirmed not to have any disease of the urinary system. The serum samples were stored at −80 °C. The specimen collection was approved by the Ethics Review Board at the Eighth Medical Center of Chinese PLA General Hospital from October 2011 to August 2014. Five‐year follow‐up of the 79 RCC patients was carried out. Written informed consent was obtained from each subject and all experiments conformed to the Declaration of Helsinki.

**Table 1:** The correlation of serum LINC00887 expression with the clinical characteristics of the RCC patients.

Variable	Serum LINC00887 expression		*P* value
	High (*n* = 39)	Low (*n* = 40)	
Age (years)			
＞ 50	20	20	0.544
≤ 50	19	20	
Gender			
Male	22	23	0.551
Female	17	17	
Lymph node metastasis			
Yes	16	14	0.375
No	23	26	
TNM stage			
T1–T2	18	20	0.454
T3–T4	21	20	

Differences among variable were analyzed using a chi‐squared test.

### Total RNA extraction

Total RNAs of tissues were isolated using a TRIzol reagent (Invitrogen, Carlsbad, CA, USA) in accordance with the manufacturer's instructions. Total RNAs in serum were extracted using a TRIzol LS reagent (Invitrogen) accordance with the manufacturer's instructions. Cel‐miR‐39, which was used to normalize LINC00887 expression in serum, was added to the samples that had been lysed by TRIzol LS reagent but before chloroform was used. Total RNAs were extracted from 250 μL of serum sample and finally dissolved using 15 µL of RNase‐free H_2_O.

### Quantitative reverse transcription‐PCR

The quantitative reverse transcriptase PCR (qRT‐PCR) experiments were performed using a QuantiTect SYBR Green RT‐PCR Kit (Qiagen, Hilden, Germany) in accordance with the manufacturer's instructions. GAPDH served as an internal control for LINC00887 expression in tissue, and cel‐miR‐39 acted as a control for LINC00887 expression in the serum. The $2^{‐\Delta \Delta C_{t}}$ method was used to calculate the relative expression of LINC00887 in tissue and serum. The primer sequences were: LINC00887 forward, 5′‐TCCTGCTTGGCAGGTAACAG‐3′, reverse, 5′‐AACGATGCCTCAGTCGAAGG‐3′; GAPDH forward, 5′‐ACCACAGTCCATGCCATCAC‐3′, reverse, 5′‐TCCACCCTGTTGCTGTA‐3′. Cel‐miR‐39 reverse transcription, 5′‐GTCGTATCCAGTGCAGGGTCCGAGGTATTCGCACTGG ATACGACCAAGCT‐3′, forward, 5′‐CAGAGTAGCTCACCGGGTGTAAATC‐3′, reverse, 5′‐CCAGTGCAGGGTCCGAGGTAT‐3′.

### Cell culture

Renal cell carcinoma cell lines including 786‐O, 769‐P, ACHN and Caki‐1 were purchased from the American Type Culture Collection (ATCC, Manassas, VA, USA). The four cell lines were cultured in Dulbecco's modified Eagle's medium (Gibco, Carlsbad, CA, USA) supplemented with 10% fetal bovine serum (Gibco) and were set in a humidified incubator with 5% CO_2_ at 37 °C.

### Transfection

ACHN and 786‐O cells were seeded in a six‐well plate and grown to 60–70% confluence. The siRNAs for LINC00887 were transfected into two RCC cell lines using a Lipofectamine 2000 reagent (Invitrogen) in accordance with the manufacturer's instructions. The siRNA sequences were: siRNA1, 5′‐GTTTCTTCTGCTTGGAACTCT‐3′; siRNA2 5′‐GCCACCATACCCAGCTCATTT‐3′.

### Cell proliferation

Twenty‐four hours after the transfection of siRNAs, ACHN and 786‐O cells were seeded in 96‐well plates at a density of 2000 per well. The cells were cultured for a further 0–5 days. Cell Counting Kit‐8 (CCK‐8; Sigma‐Aldrich, Beijing, China) reagent was used to detect cell proliferation in accordance with the manufacturer's instructions.

### Statistical analysis

All data are presented as the mean ± SD or 95% confidence interval. Comparisons between two groups were conducted using Student’s *t*‐test or a non‐parametric Mann–Whitney Wilcoxon test. Comparisons for more than two groups used one‐way ANOVA followed by Tukey’s post‐hoc test. The ROC curve was used to assess AUC values. *P* < 0.05 was considered statistically significant. spss, version 22.0 (IBM Corp., Armonk, NY, USA) and prism, version 8.0 (GraphPad Software Inc., San Diego, CA, USA) were used to analyze the data and generate graphs.

## Results

### LINC00887 expression is frequently increased in RCC tissue

**Fig. 1:**
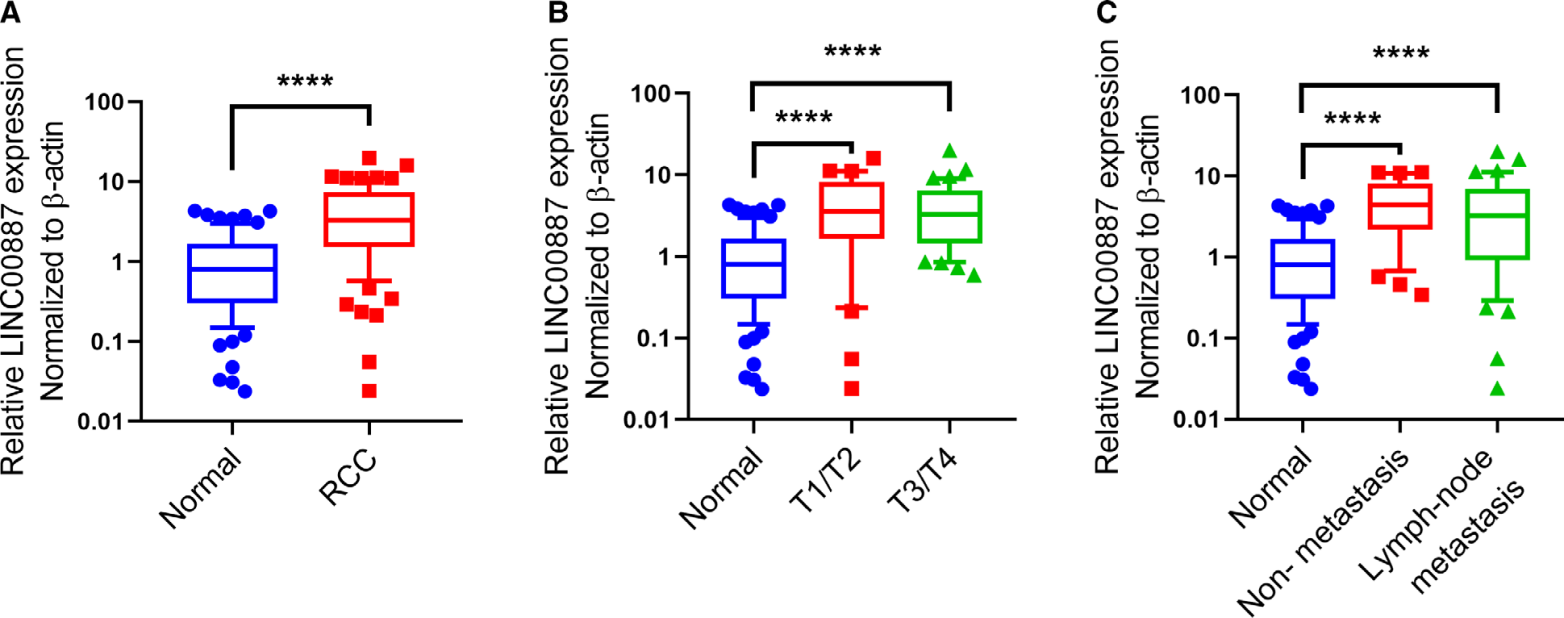
LINC00887 expression is frequently increased in RCC tissue. (A) Boxes show LINC00887 expression in 79 pairs of RCC tissues and matched normal tissues. Comparisons were performed with Student’s *t*‐test. (B) Boxes show LINC00887 expression in 38 T1/T2‐stage RCC tissues, 41 T3/T4‐stage RCC tissues and 79 matched normal tissues. (C) Boxes show LINC00887 expression in 49 lymph node‐metastatic RCC tissues, 30 non‐metastatic RCC tissues and 79 matched normal tissues. Comparisons were performed with one‐way ANOVA followed by Tukey’s post‐hoc test. LINC00887 expression was measured using qRT‐PCR, with β‐actin serving as an internal control. Whiskers: 10–90 percentile. ****P < 0.0001.

To confirm LINC00887 expression in RCC in the present study, we collected 79 pairs of RCC samples from 79 patients, including 38 patients with T1/T2‐stage RCC, 41 with T3/T4, 49 patients with lymph node metastasis and 30 without metastasis. qRT‐PCR experiments showed that LINC00887 expression was significantly increased RCC tissues compared to matched normal tissues (Fig. 1A) and also significantly increased in T1/T2‐ and T3/T4‐stage RCC tissues (Fig. 1B). However, there was no significant difference for LINC00887 expression between lymph node‐metastatic RCC tissues and non‐metastatic RCC tissues (Fig. 1C). These results suggest that LINC00887 expression is elevated in RCC tissue and may be not associated with lymph node metastasis.

### LINC00887, which is mainly derived from RCC tissues, is upregulated in the serum of RCC patients

**Fig. 2:**
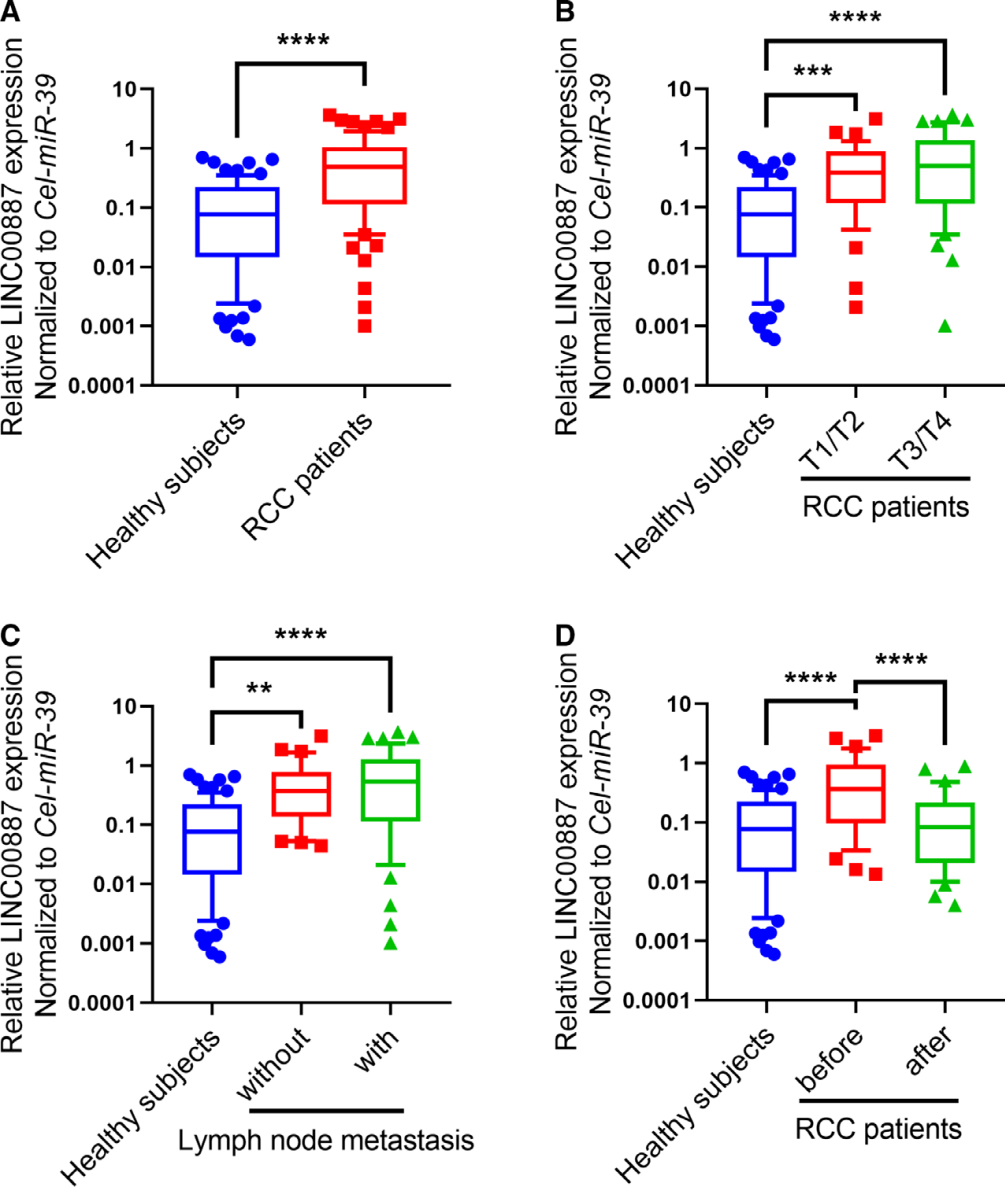
LINC00887, which is mainly derived from RCC tissues, is upregulated in the serum of RCC patients. (A) Boxes show LINC00887 expression in the serum of 79 RCC patients and 79 healthy subjects. Comparison were performed with a non‐parametric Mann–Whitney Wilcoxon test. (B) Boxes show LINC00887 expression in the serum of 38 T1/T2‐stage RCC patients, 41 T3/T4‐stage RCC patients and 79 healthy subjects. (C) Boxes show LINC00887 expression in 49 lymph node‐metastatic RCC patients, 30 non‐metastatic RCC patients and 79 healthy subjects. (D) Boxes show LINC00887 expression in the serum of 35 RCC patients before and after operation, and 79 healthy subjects. Comparisons were performed with one‐way ANOVA followed by Tukey’s post‐hoc test. LINC00887 expression was measured using qRT‐PCR, with *cel‐miR‐39* serving as an internal control. Whiskers: 10–90 percentile. **P < 0.01; ***P < 0.001; ****P < 0.0001.

As a result of the increased expression of LINC00887 in early‐stage RCC tissue, we aimed to determine whether it could be a non‐invasive biomarker for the detection of early‐stage RCC. We thus detected LINC00887 expression in serum samples from RCC patients. qRT‐PCR experiments revealed that LINC00887 expression was significantly increased in the serum of RCC patients, and also significantly increased in the serum of T1/T2‐ and T3/T4‐stage RCC patients, compared to healthy subjects (Fig. 2A), whereas there was no significant difference for serum between T1/T2‐stage and T3/T4‐stage RCC patients (Fig. 2B). Moreover, LINC00887 expression was significantly elevated in the serum of both RCC patients with and without lymph node metastasis compared to healthy subjects, whereas there was no significantly different for serum between lymph node‐metastatic and non‐metastatic patients (Fig. 2C). Next, we aimed to identify whether LINC00887 in serum is mainly derived from RCC tissues. We detected LINC00887 expression in 70 serum samples that were collected from 35 RCC patients before and after operation, respectively. qRT‐PCR experiments revealed that LINC00887 expression was significantly decreased in serum when the RCC had been removed, and the decreased expression levels were similar to those in healthy subjects (Fig. 2D). These results suggest that LINC00887 expression is elevated in the serum of RCC patients and is mainly derived from RCC.

### Serum LINC00887 signature may serve as a non‐invasive biomarker for the detection of early‐stage RCC and is associated with RCC patient survival

**Fig. 3:**
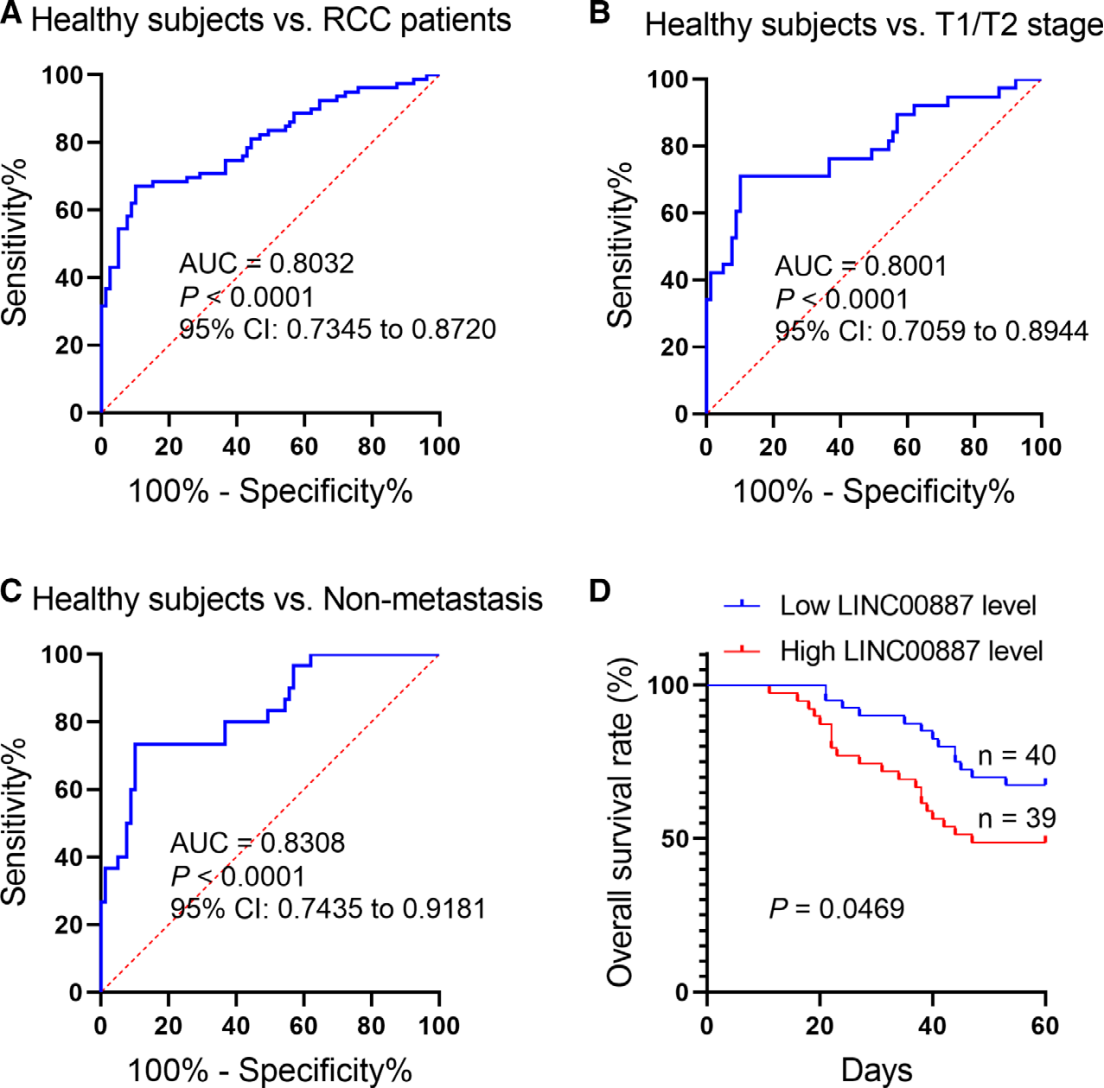
The serum LINC00887 signature may serve as a non‐invasive biomarker for the detection of early‐stage RCC and is associated with RCC patient survival. (A) ROC curve shows an AUC of serum LINC00887 signature with respect to differentiating RCC patients from healthy subjects, with a sensitivity of 67.09% and a specificity of 89.87%. (B) ROC curve shows an AUC of the serum LINC00887 signature with respect to differentiating T1/T2‐stage RCC patients from healthy subjects, with a sensitivity of 71.05% and a specificity of 89.87%. (C) ROC curve shows an AUC of serum LINC00887 signature with respect to comparing non‐metastatic RCC patients with healthy subjects, with a sensitivity of 73.33% and a specificity of 89.87%. (D) Overall survival analysis reveals RCC patients with a high expression of LINC00887 in serum have worse survival rates compared to those with low expression.

Next, we aimed to estimate the detective efficiency of the serum LINC00887 signature for early‐stage RCC. ROC analyses showed that the serum LINC00887 signature had an AUC of 0.8032 with respect to differentiating RCC patients from healthy subjects, with a sensitivity of 67.09% and a specificity of 89.87% (Fig. 3A); an AUC of 0.8001 with respect to differentiating T1/T2‐stage RCC patients from healthy subjects, with a sensitivity of 71.05% and a specificity of 89.87% (Fig. 3B); and an AUC of 0.8308 with respect to comparing non‐metastatic RCC patients with healthy subjects, with a sensitivity of 73.33% and a specificity of 89.87% (Fig. 3C). Furthermore, we analyzed the correlation of serum LINC00887 expression with the RCC patient survival. The RCC patients were divided into two groups based on the median expression of LINC00887 in the serum of the RCC patients. Overall survival analysis revealed that RCC patients with high LINC00887 expression had poorer survival rates than those with low (Fig. 3D). These suggest that serum LINC00887 signature may be a potential non‐invasive biomarker for the early detection and prognosis of RCC patients.

### LINC00887 promotes RCC cell proliferation *in vitro*

**Fig. 4:**
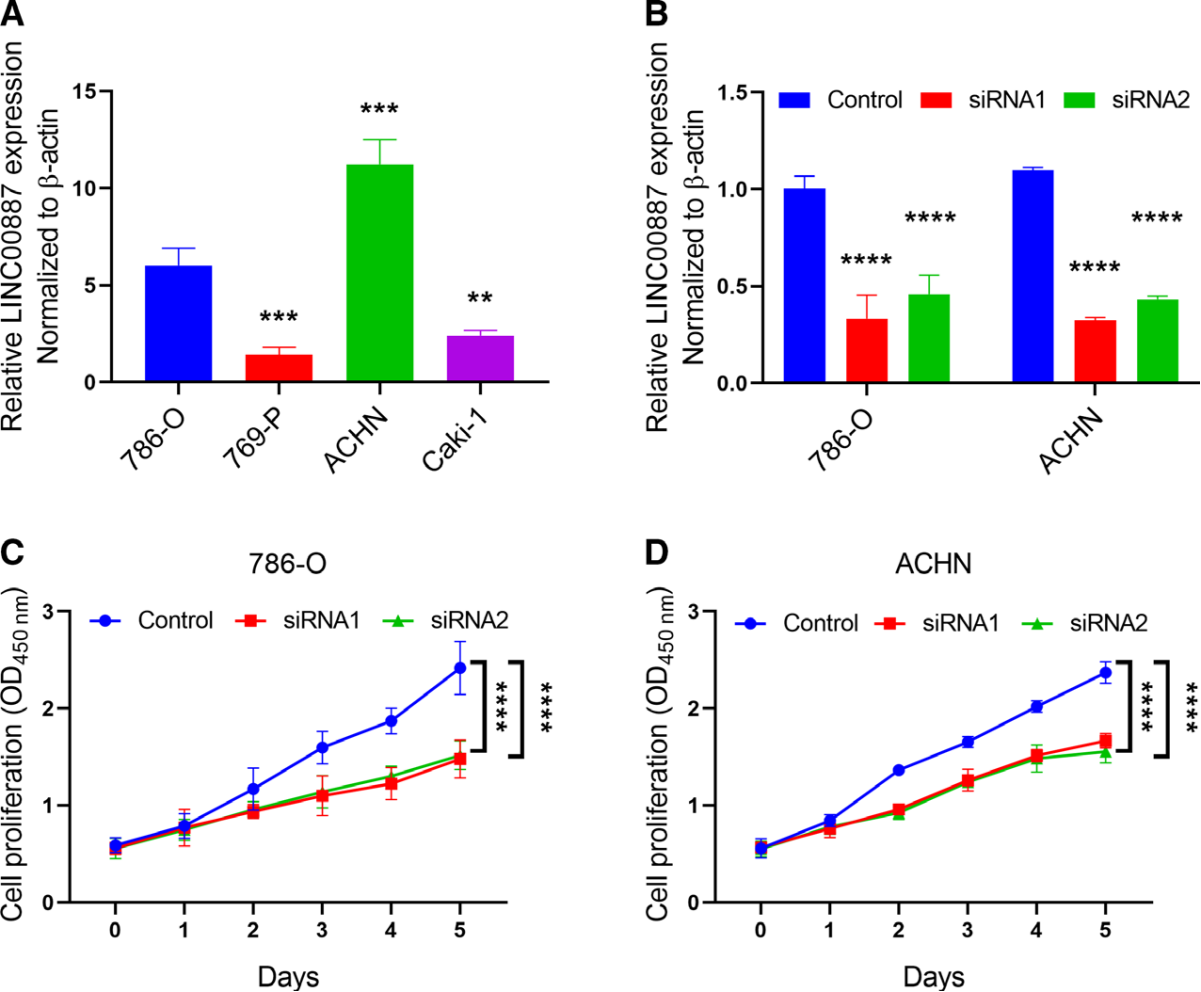
LINC00887 promotes RCC cell proliferation *in vitro*. (A) Bars show LINC00887 expression in four RCC cell lines. (B) Bars show LINC00887 expression in 786‐O and ACHN cells, which were transfected with control, siRNA1 or siRNA2, respectively. LINC00887 expression was measured using qRT‐PCR, with β‐actin serving as an internal control. Comparison were performed with Student’s *t*‐test. Data are presented as the mean ± SD. Lines reflect the proliferation of 786‐O (C) and ACHN (D) cells at different time points over 0–5 days. The two cells were transfected with control, siRNA1 or siRNA2, respectively. CCK‐8 reagent was used to analyze cell proliferation. Comparison were performed with one‐way ANOVA followed by Tukey’s post‐hoc test. All experiments were repeated three times. Data are presented as the mean ± 95% confidence interval. **P < 0.01; ***P < 0.001; ****P < 0.0001.

To explore the biological function of LINC00887 in RCC, we selected four RCC cell lines, including 786‐O, 769‐P, ACHN and Caki‐1. We measured LINC00887 expression in the four cell lines and found that ACHN cells expressed the highest level of LINC00887 compared to the other cell lines, and 786‐O cells ranked second (Fig. 4A). Therefore, we chose ACHN and 786‐O cell lines to perform the loss of function of LINC00887. qRT‐PCR experiments confirmed that both siRNA1 and siRNA2 can significantly inhibit LINC00887 expression in ACHN and 786‐O cells (Fig. 4B). Because LINC00887 expression is not associated with TNM stage or lymph node metastasis of RCC, we directly analyzed its effect on RCC cell proliferation. Cell proliferation experiments showed that LINC00887 knockdown significantly suppressed the abilities of 786‐O and ACHN cells to proliferate, respectively (Fig. 4C,D). These results suggest that LINC00887 promotes RCC cell proliferation.

## Discussion

Improving the approaches for the detection of early‐stage RCC can screen out more patients who have not developed advanced‐stage RCC. However, current non‐invasive biomarkers lack sufficient accuracy for the detection of early‐stage RCC [4]. lncRNAs may be a potential non‐invasive biomarker for cancer diagnosis on account of their organ‐specific characteristics. In recent years, serum lncRNAs have been shown to be dysregulated in cancer patients compared to healthy subjects [10, 11, 12], including RCC patients [13]. In the present study, we found a novel lncRNA, LINC00887, for which the expression is increased in both tumor tissues and the serum of early‐stage RCC patients.

Serum lncRNAs can be derived from various human organs. To confirm whether serum LINC00887 is generated from RCC tissues, we collected 35 pairs of serum samples from 35 RCC patients before and after operation, respectively. We found that LINC00887 expression was decreased in the serum of RCC patients when the RCC had been removed, suggesting that the increased level of LINC00887 in serum is involved in RCC. It is important to identify the source of serum lncRNAs. For example, Tan *et al*. [14] also detected lncRNA HOTAIR expression in both serum and tissue samples when determining the correlation between its expression in serum and tumor tissue. Our findings suggest that LINC00887 in the serum of RCC patients is derived from RCC tissue, for which a high level may reflect RCC tumorigenesis. Next, a ROC curve was generated to assess the diagnostic power of serum LINC00887. An AUC of 0.8001 was obtained with respect to differentiating T1/T2‐stage RCC patients from healthy subjects, with a sensitivity of 71.05% and a specificity of 89.87%, suggesting that serum LINC00887 signature may be a potential non‐invasive biomarker for the detection of early‐stage RCC patients.

lncRNAs have important biological functions in cancer development [15, 16, 17]. We are the first to demonstrate that LINC00887 promotes RCC cell proliferation *in vitro*. We used two effective siRNAs to knock down LINC00887 expression in an RCC cell line, ACHN, which expresses the highest level of LINC00887 compared to the other three cell lines (786‐O, 769‐P and Caki‐1) included in the present study. Next, we measured the influence of LINC00887 on RCC cell proliferation only using CCK‐8 reagent because our data indicate that LINC00887 expression is not associated with lymph node metastasis. Indeed, whether LINC00887 really has no a role in RCC metastasis requires further verification in future studies.

In conclusion, we found an upregulated lncRNA LINC00887 in tumor tissues and serum of RCC patients and its serum signature may be a potential biomarker for the detection of early‐stage RCC. We also demonstrated that LINC00887 can promote RCC cell proliferation, suggesting that LINC00887 may be a therapeutic target for RCC growth.

## Conflict of interests

The authors declare that they have no conflicts of interest.

## Author contributions

GL and CL designed this study. JX and GL wrote the manuscript and analyzed the data. JX, YZ, RC, GL, and YL performed the experiments. JX, JY, ZS, YL and YS analyzed the data. NW, JA and CL collected the RCC tissue samples and cultured the cell lines.

## Data Availability

The data are available from the corresponding author upon reasonable request.
